# Editorial: Synovial tissue biopsy research

**DOI:** 10.3389/fmed.2022.1004029

**Published:** 2022-10-04

**Authors:** Douglas J. Veale, Gary S. Firestein, Mihir D. Wechalekar, Aurélie Najm

**Affiliations:** ^1^Department of Rheumatology, University College Dublin, Dublin, Ireland; ^2^Centre for Arthritis and Rheumatic Diseases, Saint Vincent's University Hospital, Dublin, Ireland; ^3^Department of Rheumatology, University of California, San Diego, San Diego, CA, United States; ^4^Department of Rheumatology, Flinders University, Flinders Medical Centre, Adelaide, SA, Australia; ^5^Department of Rheumatology, University of Glasgow, Glasgow, United Kingdom

**Keywords:** rheumatoid arthritis, Psoriatic arthritis, synovial tissue, biopsies, cellular and molecular analysis

Rheumatoid arthritis (RA) is an inflammatory autoimmune disease, that left untreated results in pain, joint damage, and functional disability for the patients (1). In healthy persons, the synovium is a loose connective tissue with one or two cells lining the surface without a basement membrane (2). It adheres to the internal joint capsule, which is comprised of ligaments. In RA, the synovium expands with finger-like projections (villi) that are highly vascularised with new blood vessels and abundant activated immune cells including macrophages, lymphocytes, and resident stromal cells, i.e., fibroblasts and dendritic cells (3).

The synovium is the primary target tissue in RA, although research has focused on related tissue in lymph nodes as the synovium adopts many features of lymphoid tissue. The “Synovial Tissue Biopsy Research” topic focuses on highly specialized and significantly advanced “omic” technology that can be applied to small fragments of synovial tissue. The contributors are all key opinion leaders with significant experience in this topic. While the understanding of the physiological function and pathological responses of synovial tissue has advanced, several key questions remain unanswered—When and where does RA begin? What drives the immunological changes? Can synovial tissue cellular and molecular analysis reveal who is at risk or who will respond to therapies and what is the correct therapy for the correct patient?

In the paper from Anaparti et al., the synovial transcriptome is examined in early inflammatory arthritis patients (EIA) before treatment to identify potential biomarkers of long-term outcomes of RA. Synovial biopsies were obtained from clinically inflamed knee joints, using a needle biopsy, prior to disease-modifying anti-rheumatic drug (DMARD) therapy or from advanced RA patients undergoing joint replacement. Affymetrix Human Genome U133 Plus 2 microarray platform was used and clinical follow-ups collected metadata for over a decade. Short- and long-term outcomes were determined by RA-associated clinical parameters. A number of differentially expressed genes (DEGs), including key matrix metalloproteinases (MMP)-1 and MMP-3 were identified in DMARD-naïve EIA patients compared to advanced RA patients. Interestingly, MMP-3 has previously been described as a marker of subsequent bone erosions in early RA (4). Subjects were defined as MMP-high or MMP-low, on RNA and protein analysis (qPCR and immunohistochemistry) and hierarchical clustering identified 947 DEGs between MMP-high and MMP-low cohorts. The MMP-high cohort showed an enrichment of genes associated with metabolic/biochemical functions and intracellular signaling regulated particularly by NF-κB and β-catenin complexes and correlated with systemic inflammatory markers. In the short-term, the MMP-high cohort showed a significant reduction in disease activity scores, modified HAQ, and correlated negatively with baseline MMP-1. They conclude that gene expression profiling of synovial tissue biopsies allows the identification of patient subsets according to disease activity and may predict long-term clinical outcomes.

O'Byrne et al. highlight RA pathogenesis linking the immunological changes in synovial tissue biopsies and lymph nodes. Autoantibodies associated with RA (rheumatoid factor & anti-citrullinated peptide antibodies) may be found in the circulation several years before joint swelling/tenderness or evidence of inflammation is detected clinically. This suggests a break in immunological tolerance precedes disease onset. The authors describe studies exploring secondary lymphoid organs for evidence of an initial break in peripheral tolerance. If explored during the earliest phases of RA, lymph node research may provide innovative drug targets for disease modulation or prevention. RA research largely centers on the role and origin of lymphocytes and macrophages that infiltrate the joint, as well as growing efforts to determine the role of stromal cells within the synovium. This research examines subsets and activation of T cells, dendritic cells, and stromal cells in lymph nodes as animal studies suggest a prominent immunomodulatory role for lymph node stromal cells. O'Byrne et al. suggest that synovium and proximal peripheral lymph nodes should be investigated in conjunction with one another to gain an understanding of the immunological processes driving RA progression from systemic autoimmunity toward synovial inflammation.

The contribution by Floudas et al. focuses on novel techniques that may be useful to interrogate the immunological changes in synovial tissue biopsies as the target tissue in RA and Psoriatic arthritis (PsA). Immune and stromal cells are in a highly activated state within the inflamed joint and cell interactions may provide vital clues to the pathogenesis. However, research into synovial tissue presents significant technical difficulties due to the size and quality of the tissue and the sampling methods. The authors highlight the importance of advances in high-throughput technologies developed to optimize the information gained from such studies. They address methodological challenges in the preparation and processing of synovial tissue, the effect of storage and handling techniques on downstream analysis, and potential synergies between such complex analyses. The focus is on advances in high dimensionality flow cytometric analysis, single-cell RNA sequencing in parallel with functional assays *ex vivo*, and non-intrusive metabolic characterization of synovial cells on a single cell level based on fluorescent lifetime imaging microscopy. The authors highlight the importance of, and how to implement, novel research techniques that will accelerate our understanding of the pathogenesis of RA and PsA which may allow the discovery of novel therapeutics.

Lipidomics, a specialized division of metabolomics, allows the identification and quantification of lipids in biosamples and has been optimized for synovial tissue biopsies by Coras et al. Lipidomics offers the potential for the wide-ranging categorization of cell types involved in RA pathogenesis and their associated functional metabolomics. Studies performed to date have defined synovial pathotypes related to disease severity and treatment response. The authors discuss data from animal models and human biosamples from inflammatory arthritis patients measuring glycerophospholipids, glycerolipids, sphingolipids, oxylipins, and fatty acids implicated in arthritis pathogenesis. They discuss the literature on lipid quantification in synovial tissue biopsies and how these impact the inflammatory pathways. Finally, they outline how integrating lipidomics along with other “omic” technologies to analyse synovial tissue biopsies will advance our understanding of the mechanisms at play in the synovial inflammatory lesions and may also enable the development of new therapeutic strategies.

As outlined in several of the contributions to this Research Topic, the synovial tissue is an immunologically complex environment involving the interaction of diverse and highly specialized subsets of immune cells. Knab et al. outline the different functions that some of these cells appear to exhibit. Some are pro-inflammatory, others regulatory and then in some patients, they explain that resident synovial tissue cells develop an aggressive, invasive phenotype that may lead to cartilage or bone damage. They describe how the balance between protection and damage is in constant flux, with macrophage and fibroblast cells predominantly working to prevent synovial tissue inflammatory danger signals produced by the cartilage and synovial fluid in response to mechanical stress. In this review, the authors discuss the molecular characteristics of synovial cells resident in the synovial tissue, mainly macrophages and fibroblasts, and how the crosstalk between these cells may impact homeostasis and inflammation in joint diseases such as rheumatoid arthritis.

Boyle et al. describe the synovial microenvironment and how synovial tissue biopsy research has increased our understanding of the complex cellular interplay between resident cells and infiltrating immune cells. The authors describe the methods they have optimized to study synovial tissue biopsies, in particular the process used to disaggregate the synovial tissue to yield the best results for applying “omic” technologies such as single-cell RNA sequencing. The authors used synovial tissue biopsies obtained at arthroplasty from patients with RA and control subjects with osteoarthritis. The synovial tissue biopsies were then processed using different methods to compare the fidelity of the RNA extraction. The study outcomes included the cell yield and viability, in addition to, the candidate gene analysis which was dramatically increased using the current standard Liberase method. They show that the disaggregation techniques used for connective tissues, including synovial tissue biopsies, may have complicated specific influences on the transcriptome. Their findings confirm that the use of an RNA polymerase inhibitor or a cold enzyme in the disaggregation process limits the induction of changes to the transcripts as a result of processing. Boyle et al. concludes that by using these updated processes the output from the disaggregated transcriptome is a better reflection of the in situ transcriptome. They also highlight that a specific methodology may be used to provide optimal outputs depending on the specific genes being studied and the hypothesis being tested.

## Author contributions

All authors listed have made a substantial, direct, and intellectual contribution to the work and approved it for publication.

## Conflict of interest

The authors declare that the research was conducted in the absence of any commercial or financial relationships that could be construed as a potential conflict of interest.

## Publisher's note

All claims expressed in this article are solely those of the authors and do not necessarily represent those of their affiliated organizations, or those of the publisher, the editors and the reviewers. Any product that may be evaluated in this article, or claim that may be made by its manufacturer, is not guaranteed or endorsed by the publisher.
